# Clinical Outcome of the Oblique Locking Hip Screw

**DOI:** 10.1155/aort/5082003

**Published:** 2025-11-17

**Authors:** Shota Nakagawa, Masato Toyonaga, Takeshi Sawaguchi, Takashi Matsushita

**Affiliations:** ^1^ Trauma and Reconstruction Center, Shin-yurigaoka General Hospital, Kawasaki, Japan, shinyuri-hospital.com; ^2^ Department of Traumatology, Fukushima Medical University, Fukushima, Japan, fmu.ac.jp

**Keywords:** compression hip screw, Oblique Locking Hip Screw, sliding hip screw, trochanteric fractures

## Abstract

**Purpose:**

To evaluate the clinical outcomes and effectiveness of the newly developed Oblique Locking Hip Screw (OLHS) compared with those of the commonly used cephalomedullary nail (CMN) in trochanteric femoral fracture treatment in older patients.

**Methods:**

This was a single‐center retrospective study of patients with trochanteric fractures. Overall, 129 patients were analyzed: 64 treated with OLHS and 65 with CMN. Patient demographic data, fracture classification, surgical parameters (e.g., operating time and intraoperative blood loss), and clinical outcomes were assessed. Postoperative outcomes were evaluated using radiographic findings and statistical analyses, including Fisher’s exact test and the Mann–Whitney *U* test.

**Results:**

The study included 57 patients in both the OLHS and CMN groups with 3 months of radiographic follow‐up data. OLHS was more commonly used for stable AO A1 fractures, whereas CMN was preferred for unstable A2 fractures. Postoperative telescoping was greater with OLHS than with CMN (4.8 ± 4.3 vs. 3.0 ± 4.7 mm; *p* = 0.0028; mean difference 1.8 mm, 95% CI 0.15–3.45). Mortality, union, and adverse events were similar; two CMN patients had nonunion, with one conversion to total hip arthroplasty.

**Conclusion:**

OLHS provides satisfactory clinical outcomes for trochanteric fractures in this exploratory study, with preliminary evidence of adequate stability. Further randomized controlled trials or matched comparative studies are warranted to confirm these findings.

## 1. Introduction

Hip fractures are common and can be life‐threatening in older populations. Implants used for the treatment of trochanteric fractures are generally categorized into two types: extramedullary devices (sliding hip screw [SHS]/compression hip screw [CHS]) and intramedullary devices (cephalomedullary nail [CMN]/intramedullary nail [IN]).

The guidelines from the Japan Orthopaedic Association state that although CMN has some advantages for unstable fractures, there is no clinical difference between the use of CMN and SHS for all trochanteric fractures [1].

Conversely, surgeons in most institutions in Japan use CMN regardless of the fracture type, with approximately 90% of all trochanteric fractures treated with CMN [2].

However, SHS still offers some advantages over CMN. First, SHS is less expensive than CMN, with many studies showing its cost‐effectiveness [3–5]. Second, SHS can be inserted with the hip in the valgus position, facilitating easier valgus fracture reduction. Notably, valgus reduction is preferred over varus reduction. Marmor et al. [6] compared both reduction alignments and concluded that avoiding varus reduction can reduce implant loading. In addition, the valgus hip position facilitates improved surgical access in patients with obesity, short legs, and wide iliac wings. Finally, SHS can be used for the treatment of patients with narrow femoral canals. However, although SHS has several advantages, it lacks the stability required for the treatment of unstable fractures.

We introduced a new type of SHS, the Oblique Locking Hip Screw (OLHS; HOYA Technosurgical Corporation, Tokyo, Japan), designed to enhance constructional rigidity and rotational stability through additional proximal and distal locking pins.

We primarily use OLHS for stable Arbeitsgemeinschaft für Osteosynthesefragen (AO) A1 fractures, as lateral wall thickness is a key factor in the failure of SHS implants [7]. However, the specific characteristics of the OLHS may offer advantages in the treatment of unstable fractures.

In 2002, Ikuta introduced a classification system for the postoperative lateral view (three views) in the Japanese literature [8], which was later expanded by Utsunomiya in 2004 to include the classification of the anterior–posterior view [9]. Since then, the importance of reduction quality, with a focus on anteromedial cortical support, has been widely upheld in Japan. In 2015, Chang et al. modified the Baumgartner classification [10, 11] by focusing on positive medial cortical support [12]. Our hypothesis is that a high‐rigidity SHS implant, specifically the OLHS, with adequate anteromedial cortical support, can fix both stable and unstable fractures. To assess our hypothesis, this study was conducted to analyze the clinical outcomes and efficacy of the OLHS and compare them with those of the CMN as a reference group.

A preliminary version of this work was previously posted as a preprint on Research Square [13].

The present study builds upon and expands that preliminary report by including updated analyses and clarifications in accordance with peer review standards.

## 2. Materials and Methods

### 2.1. Study Design and Patient Selection

This was a single‐center, retrospective study of patients with trochanteric femoral fractures who underwent surgery between April 2020 and December 2022. Eligibility criteria were age ≥ 60 years, an isolated trochanteric femoral fracture, and treatment with either the OLHS or a CMN. Exclusion criteria were a combination of a completely lateral wall fracture (AO 31A3 [14]), high‐energy trauma, pathological fractures, periprosthetic fractures, and clinical/radiographic follow‐up shorter than 3 months.

**Figure 1:**
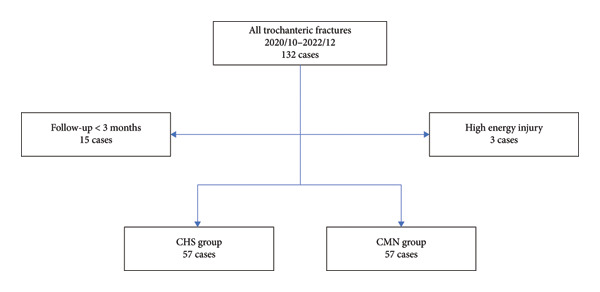
Flowchart of patient selection. Of 132 consecutive cases, 3 were excluded because of high‐energy trauma and 15 because of follow‐up shorter than 3 months, resulting in 114 patients included in the final analysis (OLHS: 57; CMN: 57).

Of 132 eligible cases, 3 were excluded for high‐energy trauma and 15 for < 3 month follow‐up, leaving 114 patients for analysis (OLHS *n* = 57; CMN *n* = 57). Within the CMN cohort, 30 short nails and 27 long nails were used. The patient selection process is summarized in Figure 1.

Data on the patients’ age, sex, and body mass index (BMI) were assessed. In addition, the patients’ preoperative conditions were evaluated using the American Society of Anesthesiologists Physical Status classification [15]. Furthermore, the patients were classified using initial radiographs (X‐rays) and CT scans according to the AO, Li et al. [16], and Nakano 3DCT classifications [17]. Data relevant to the operation (surgical time and intraoperative blood loss) were also assessed.

### 2.2. Description of the OLHS

The OLHS; HOYA Technosurgical Corporation, Tokyo, Japan is a newly developed extramedullary fixation device designed to provide greater rigidity and rotational stability compared with conventional compression hip screws.

The OLHS system consists of the following components (Figures 2(a) and 2(b)):

1. A lag screw inserted along the femoral neck axis and housed within a 12‐mm barrel on the side plate;
2. Two proximal smooth pins (5.0 mm in diameter, 30–75 mm in length) placed parallel to the lag screw to prevent rotation of the femoral head and neck fragment; and
3. Three distal smooth pins for shaft fixation—one horizontal and two oblique—each measuring 4.2 mm in diameter and 30–70 mm in length.

Figure 2(a) Oblique Locking Hip Screw. (b) Smooth pins. Left is smooth pin for proximal part; right is smooth pin for distal part. (c) Integrated triple drill guide to insert guide pins from femur shaft to neck.

**Figure:**
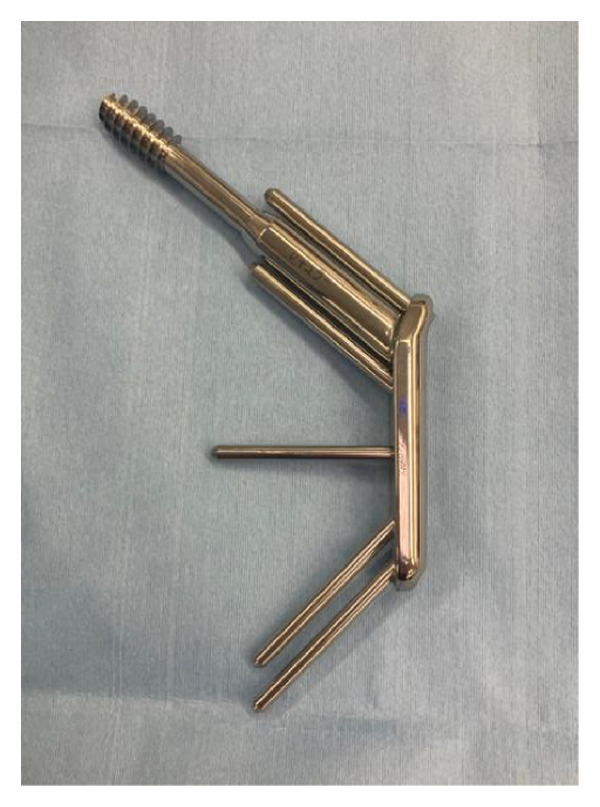
(a)

**Figure:**
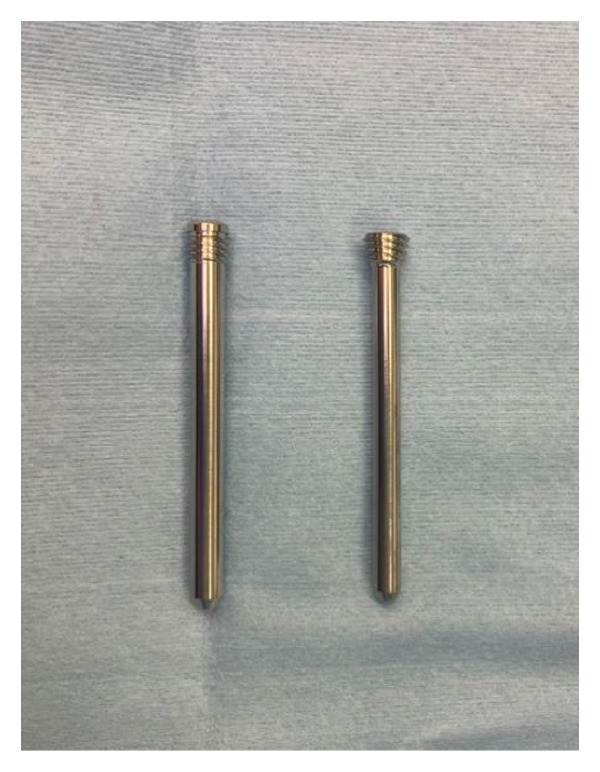
(b)

**Figure:**
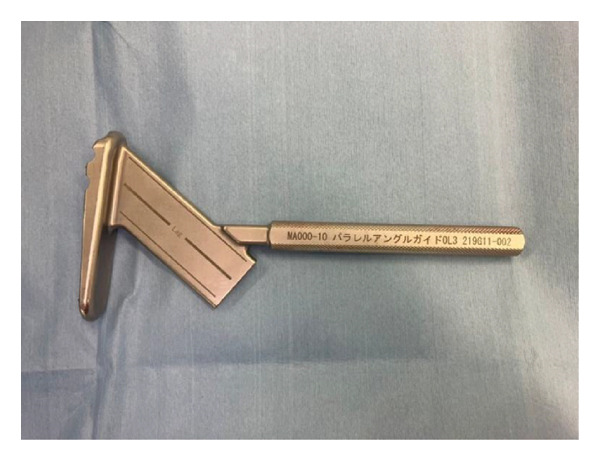
(c)

The side plate has a thickness of 8.2 mm and a width of 19 mm and allows oblique insertion of the distal pins—one horizontally and two at approximately 45° downward—to enhance constructional rigidity. The two proximal pins are oriented approximately 45° upward relative to the shaft axis.

The implant body is made of titanium alloy (Ti‐6Al‐4V) and has been approved by the Pharmaceuticals and Medical Devices Agency (PMDA), Japan. (The FDA/CE status: “not obtained”). Two plate sizes are available: small and regular.

In the small size, a combination of 65‐mm (proximal) and 75‐mm (distal) smooth pins is recommended to ensure that even at maximum telescoping, the pins do not penetrate the femoral head. In the regular plate, 75‐mm proximal pins are generally used.

Biomechanical testing has demonstrated that the OLHS can withstand cyclic loading of 800 N for 900,000 cycles without mechanical failure, confirming its high fatigue strength and antirotation capability (see Supporting Information 1 for details of the testing protocol).

### 2.3. Surgical Technique and Implant Selection

All surgeries were performed by a total of 20 orthopedic surgeons, 18 of whom were board‐certified. For every procedure, at least one trauma‐specialized orthopedic surgeon participated as the primary surgeon or assistant, ensuring standardized operative technique across all cases. Most of the surgeries were performed within 48 h after admission. Under general anesthesia or spinal anesthesia, patients were positioned on the fracture table. The hip was positioned in abduction for the OLHS and adduction for the CMN. The image intensifier used in the operation was Cios Select FD (Siemens Healthineers AG, Munich, Germany). Closed reduction was attempted initially, typically by applying traction with the hip in an abducted position, followed by internal rotation. If positive anteromedial cortical support was not achieved, we performed the Kapandji technique, inserting an elevatorium into the fracture site from the anterolateral or lateral side of the femur. After adequate reduction, we inserted the implants.

Implant selection was based on surgeon preference and fracture morphology. CMN was preferred for cases with a thin lateral wall (< 20.5 mm) or anteromedial comminution, where cortical support was insufficient. All SHS‐type implants used in this study were OLHS. The CMN models used were TFN‐ADVANCED (Johnson & Johnson MedTech, New Brunswick, USA) and INTERTAN (Smith & Nephew, Watford, UK).

### 2.4. Surgical Technique for OLHS Fixation

The OLHS procedure was performed as follows:

1. After fracture reduction, a skin incision of 8–10 cm was made from the proximal end of the femur near the greater trochanter (unnamed tubercle) along the femoral axis.
2. The iliotibial band was incised, and the vastus lateralis was split or elevated posteriorly to expose the lateral aspect of the femoral shaft.
3. Using an integrated triple drill guide (Figure 2(c)), 2.8‐mm guide pins (two to three) were inserted simultaneously through the guide into the femoral neck and head, directed toward the central axis of the neck and head in both anteroposterior and lateral views. This configuration also helped prevent rotational displacement of the femoral head during drilling for the lag screw.
4. The lag screw track was drilled and tapped, and the OLHS implant was inserted. The lag screw was advanced with a screwdriver, and the side plate was compressed against the femoral cortex using an impactor.
5. To prevent plate rotation, the distal portion was temporarily fixed with a 2.0‐mm Kirschner wire.
6. The proximal locking pins were inserted first, followed by the horizontal distal pin after drilling through the plate holes.
7. The temporary K‐wire was then removed, and two oblique distal locking pins were inserted.
8. The wound was closed layer by layer, and full weight bearing was allowed immediately after surgery.

### 2.5. Postoperative Assessment

Patients without 3‐month radiographs (*n* = 15) were excluded; baseline characteristics were similar to those of the included patients, minimizing attrition bias. Postoperative reduction was assessed using the initial postoperative radiographs. In Japan, the Utsunomiya classification [10] is commonly used for anterior–posterior radiographic assessment, whereas the Ikuta classification [9] is used for lateral view assessment. In this study, we incorporated the Baumgaertner [11, 12] and Chang classifications [13] for further assessment. Bone union was assessed using the final set of X‐rays. We defined bone union as at least three out of four sides showing union by anterior–posterior and lateral views. In addition, according to the Radiographic Union Score for Hip fracture (RUSH) [18], bone union corresponds to a RUSH score of ≥ 13, whereas nonunion corresponds to a RUSH score of ≤ 8 after 6 months postoperatively.

### 2.6. Radiographic Measurements

The tip–apex distance and telescoping were measured on initial postoperative X‐rays and adjusted based on the diameter of the lag screw. Telescoping was measured as the change in the distance from the tip of the lag screw to the lateral edge of the implant between the immediate postoperative and final follow‐up radiographs, adjusted for the known diameter of the lag screw to correct for magnification. All measurements were performed by a single board‐certified orthopedic surgeon specializing in trauma care with over 10 years of clinical experience. To ensure consistency, the same observer repeated the measurements twice at different times, and the mean values were used for analysis. Inter‐ and intraobserver reliability was not statistically assessed because all measurements were performed by a single experienced observer; however, repeated measurements were highly consistent.

### 2.7. Ethics Approval

This study was approved by the Institutional Review Board of Shin‐yurigaoka General Hospital (Approval no. 2024‐12‐1, approved on December 2, 2024). All procedures were performed in accordance with the principles of the Declaration of Helsinki.

### 2.8. Statistical Analyses

Statistical analyses were primarily performed using R software (Version 4.05; https://www.r-project.org). Because this study represents the first clinical assessment of OLHS, a priori sample size calculation was not performed. Fisher’s exact test was used to compare categorical variables, including sex, ASA Physical Status (ASA‐PS), fracture classification (AO, Tang, and Nakano 3DCT), reduction quality, presence of cases with a tip–apex distance > 25 mm, mortality, bone union, nonunion, and postoperative complications.

The Mann–Whitney *U* test was used to compare continuous variables, including age, BMI, follow‐up duration, operative time, intraoperative blood loss, and telescoping. Statistical significance was set at *p* < 0.05, and all tests were two‐tailed. Effect sizes and 95% confidence intervals for continuous outcomes were calculated using SPSS Statistics software (Version 29.0.2.0; IBM Corp., Armonk, NY, USA). Given the limited sample size, this study may not have sufficient statistical power to detect small between‐group differences. Given the exploratory nature of this study, no adjustment for multiple comparisons was applied.

### 2.9. Subgroup Analyses

In addition to the overall comparison, subgroup analyses were performed according to the fracture subtype.

Patients were stratified into AO 31A1 (stable) and AO 31A2 (unstable) fractures, and outcomes were compared between the OLHS and CMN groups within each subtype.

A further exploratory analysis compared outcomes between short and long CMN constructs. These analyses were conducted using the same statistical methods as the main analysis, and the results are presented in Supporting Tables S1–S3.

## 3. Results

### 3.1. Patient Demographics and Fracture Characteristics

The patient demographic data and fracture types are presented in Table 1. The OLHS group included 13 males and 44 females, with a mean age of 86.1 ± 8.4 years. The CMN group comprised nine males and 48 females, with a mean age of 84.8 ± 9.4 years. The BMI of the patients in the OLHS group was 19.7 ± 3.3, whereas that of those in the CMN group was 20.6 ± 3.5. The mean clinical follow‐up duration was 31.5 ± 17.5 months for the OLHS group and 38.4 ± 21.4 months for the CMN group. The mean duration of hip radiographic follow‐up was 16.0 ± 14.0 months for the OLHS group and 18.4 ± 16.0 months for the CMN group. The average operating time was 73.6 ± 25.7 min for the OLHS group and 79.3 ± 48.2 min for the CMN group. Mean intraoperative blood loss was 67.0 ± 89.9 cc for the OLHS group and 83.4 ± 140.6 cc for the CMN group. Regarding preoperative patient conditions, 0 patients in the OLHS group and three patients in the CMN group had an ASA classification of PS1. In addition, 38 in the OLHS group and 40 in the CMN group had a PS2 classification, whereas 19 in the OLHS group and 14 in the CMN group had a classification of PS3. There were no statistically significant differences in patient demographics between the groups.

**Table 1:** Patients’ demographic data and fracture types.

	OLHS *n* = 57	CMN *n* = 57	*p*	Difference [95% CI]
Background data				
Age (mean)	86.1 ± 8.4	84.8 ± 9.4	0.43^∗^	1.3 [−2.0, 4.7]
Sex (male/female)	13/44	9/48	0.48^∗∗^	
BMI	19.7 ± 3.3	20.6 ± 3.5	0.15^∗^	−0.9 [−2.2, 0.4]
Follow‐up period (months)				
Clinical	31.5 ± 17.5	38.4 ± 21.4	0.06^∗^	−6.9 [−14.1, 0.4]
Radiographic	16.0 ± 14.0	18.4 ± 16.0	0.40^∗^	−2.4 [−8.0, 3.2]
Operating time (min)	73.6 ± 25.7	79.3 ± 48.2	0.44^∗^	−5.7 [−20.0, 8.7]
Blood loss (cc)	67.0 ± 89.9	83.4 ± 140.6	0.46^∗^	−16.4 [−60.3, 27.5]
ASA PS			0.15^∗∗^	
PS 1	0	3		
PS 2	38	40		
PS 3	19	14		
Fracture type				
AO classification			< 0.001^∗∗^	
A1	33	14		
A2	24	43		
Tang classification			0.028^∗∗^	
Type 1	40	25		
Type 2	0	2		
Type 3	10	18		
Type 4	7	12		
Type 5	0	0		
Nakano 3DCT classification			0.029^∗∗^	
Type 1‐2	25	13		
Type 1‐3	27	32		
Type 1‐4	5	12		
Type 2	0	0		

Abbreviations: ASA PS = American Society of Anesthesia Physical Status, BMI = body mass index, CMN = cephalomedullary nail, OLHS = Oblique Locking Hip Screw.

^∗^Mann–Whitney *U* test.

^∗∗^Fisher’s exact test.

We also assessed the fracture types using the AO, Tang, and Nakano 3DCT classifications. OLHS was more frequently used for the treatment of stable AO A1 fractures (33 in the OLHS group and 14 in the CMN group), whereas unstable A2 fractures were more common in the CMN group (24 in the OLHS group and 43 in the CMN group). Analysis of the fractures according to the Tang classification revealed the following findings: Type 1, 40 in the OLHS group and 25 in the CMN group; Type 2, none in the OLHS group and two in the CMN group; Type 3, 10 in the OLHS group and 18 in the CMN group; Type 4, seven in the OLHS group and 12 in the CMN group; and Type 5, none in both groups. For the Nakano 3DCT classification, the findings were as follows: Type 1‐2, 25 in the OLHS group and 13 in the CMN group; Type 1‐3, 27 in the OLHS group and 32 in the CMN group; and Type 1‐4, five in the OLHS group and 12 in the CMN group. The results of Fisher’s exact test indicated that the two groups exhibited significant differences in the AO, Tang, and Nakano 3DCT classifications (*p* < 0.05).

### 3.2. Radiographic Assessment and Reduction Quality

We assessed postoperative reduction in the present study (Table 2). Evaluation using the Utsunomiya classification indicated that each group had two patients with the intramedullary type. The extramedullary valgus type was the most common type and was observed in 55 patients in the OLHS group and 54 in the CMN group. The extramedullary varus type was absent in the OLHS group but present in one patient in the CMN group. Analysis according to the Ikuta classification showed that 19 and 14 patients in the OLHS and CMN groups, respectively, had Subtype A reduction. Subtype N was observed in 30 and 39 patients in the OLHS and CMN groups, respectively. Evaluation using the Baumgaertner classification showed good reduction results in both groups: “Good” in 49 patients in the OLHS group and 51 in the CMN group; “Acceptable” in six patients in each group; and “Poor” in two patients in the OLHS group.

**Table 2:** Postoperative assessment.

Postoperative assessment	OLHS	CMN	*p*	Difference [95% CI]
Utsunomiya classification			0.60^∗∗^	
Intramedullary	2	2		
Extramedullary valgus	55	54		
Extramedullary varus	0	1		
Ikuta classification			0.20^∗∗^	
Subtype A	19	14		
Subtype N	30	39		
Subtype P	8	4		
Baumgaertner classification			0.36^∗∗^	
Good	49	51		
Acceptable	6	6		
Poor	2	0		
Chang classification			0.41^∗∗^	
Excellent	47	51		
Acceptable	9	6		
Poor	1	0		
Tip‐apex distance			1.0^∗∗^	
> 25 mm	0	1		
≤ 25 mm	57	56		
Telescoping (mm)	4.8 ± 4.3	3.0 ± 4.7	0.032^∗^	1.8 [0.16, 3.5]

^∗^Mann–Whitney *U* test.

^∗∗^Fisher’s exact test.

Analysis using the Chang classification revealed good reduction results in both groups: “Excellent” in 47 patients in the OLHS group and 51 patients in the CMN group; “Acceptable” in nine patients in the OLHS group and six patients in the CMN group; and “Poor” in one patient in the OLHS group. There were no significant differences in postoperative reduction among the classifications.

The tip–apex distance was measured, with only one patient in the CMN group showing a distance exceeding 25 mm. Postoperative telescoping was greater with OLHS than with CMN (4.8 ± 4.3 vs. 3.0 ± 4.7 mm; *p* = 0.032; mean difference 1.8 mm, 95% CI 0.16–3.5).

### 3.3. Clinical Outcomes

The clinical outcomes are shown in Table 3. Only one patient in the OLHS group died within 1 month after surgery; no patient in the CMN group died within 1 month after surgery. Two patients in the OLHS group and one in the CMN group died within 3 months.

**Table 3:** Clinical outcomes.

Clinical outcomes	OLHS	CMN	*p*
Mortality			
≤ 1 month	1	0	0.50^∗^
≤ 3 months	2	1	0.60^∗^
Clinical outcomes at the last visit			
Bone union	53	53	0.14^∗^
Nonunion	0	2	0.50^∗^
Cut‐through	0	1	1.0^∗^

^∗^Fisher’s exact test.

Bone union was achieved in 53 patients each in the OLHS and CMN groups, respectively. One patient who was followed for more than 3 months died before radiographic bone union was confirmed. This case was excluded from the bone union analysis but was not classified as a nonunion. One patient in the CMN group experienced a cut‐through and required conversion to total hip arthroplasty. There were no significant differences in mortality, bone union rate, and cut‐through rate between the two groups.

### 3.4. Subgroup Analyses

In the AO 31A1 subgroup (stable fractures), no significant differences were found between OLHS and CMN in demographic, radiographic, or clinical outcomes (Supporting Table S1).

In the AO 31A2 subgroup (unstable fractures), postoperative telescoping was significantly greater in the OLHS group (5.8 ± 4.6 mm) than in the CMN group (2.9 ± 4.1 mm, *p* = 0.013, mean difference 2.9 mm, 95% CI 0.7–5.2), whereas bone union and complication rates were comparable between the groups (Supporting Table S2).

Within the CMN group, no significant differences in operative time, blood loss, or clinical outcomes were observed between short and long nail constructs (Supporting Table S3).

### 3.5. Case Presentation

An 86‐year‐old female fell and experienced pain in her left hip. The x‐ray photograph showed a left trochanteric fracture (Figure 3(a)). Lateral wall thickness measured using computed tomography indicated 31A2.3 in the AO classification (Figure 3(b)). Operation with OLHS was performed (Figure 3(c)). She became able to walk without a cane. The radiograph of her last visit at 10 months after surgery showed that good bone union was achieved with good alignment (Figure 3(d)).

Figure 3(a) X‐ray photograph at injury. Left is AP view; right is lateral view. (b) CT view. Fracture is categorized into 31A2.2. (c) Postoperative X‐ray photograph. Left is AP view; right is lateral view. (d) X‐ray photograph at 10 months after operation. Left is AP view; right is lateral view. Bone union was achieved.

**Figure:**
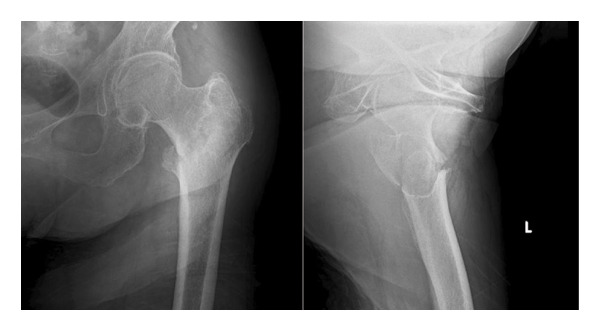
(a)

**Figure:**
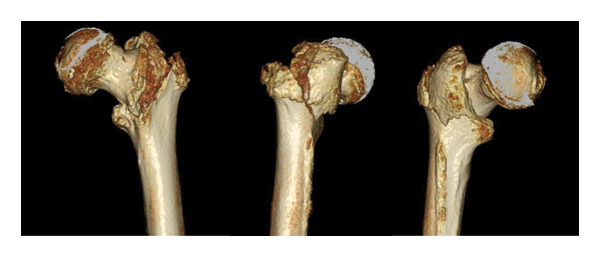
(b)

**Figure:**
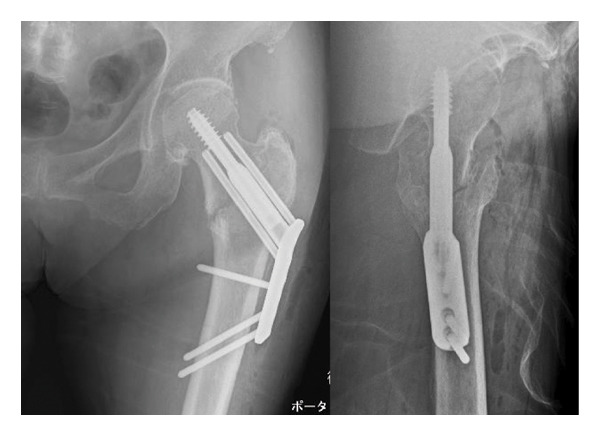
(c)

**Figure:**
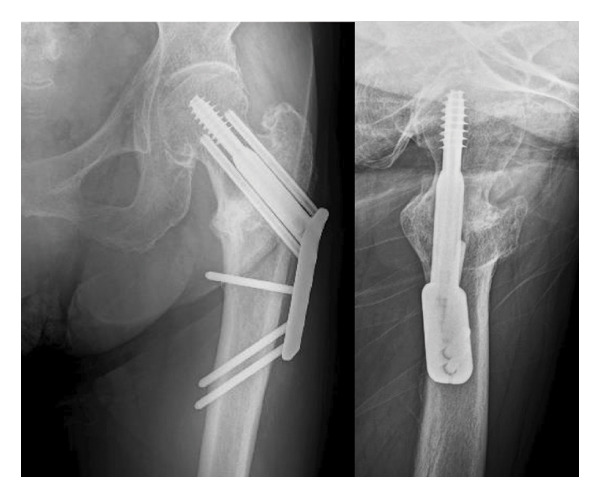
(d)

## 4. Discussion

The debate regarding implants for trochanteric fractures, CHS versus CMN, has persisted for several decades. Several reports on these implants, including randomized controlled trials, meta‐analyses, and systematic reviews, have been published. Most of these studies indicated few or no significant differences between the two implants. Specifically, many studies failed to show any differences in mortality rates between patients treated using SHS and those treated using CMN [1, 3–5]. In addition, some reports suggest that CMN is associated with less intraoperative blood loss and shorter operating time [19–21] than is SHS. Furthermore, it has been reported that SHS is associated with a higher nonunion rate than is CMN [22]; however, some studies showed no significant difference in nonunion rates between the two implants [3, 19, 23]. The reported results regarding cut‐out rates are similar to the abovementioned findings [21, 24], with fractures around the implant occurring more frequently in patients who received CMN than in those treated with SHS [19, 22, 24]. In addition, the reoperation rate for CMN is higher than that for SHS [19, 24]. Considering these complications, some researchers have suggested using intramedullary implants for unstable A2 fractures. However, SHS yields acceptable results for stable fractures [19].

In this study, we assessed OLHS compared with CMN as the reference group. Because of the retrospective study, we found that unstable fractures were more common in the CMN group.

Postoperative quality reduction was achieved in both groups. Although OLHS demonstrated slightly larger telescoping than CMN, no clinical complications such as nonunion or cut‐through were observed, suggesting that the increased telescoping did not negatively impact outcomes in this cohort. The absolute difference in telescoping between the two groups was small and unlikely to be clinically meaningful, indicating that this finding does not compromise the overall stability or functional recovery associated with OLHS fixation. The high rigidity OLHS with acceptable reduction quality may contribute to our results. The findings of another study conducted in Japan support this hypothesis [25]. These results indicate that OLHS is beneficial for managing all trochanteric fractures.

These subgroup analyses support the robustness of the overall findings, indicating that OLHS achieved comparable bone healing and complication rates to CMN across both stable (A1) and unstable (A2) fracture patterns. In the AO 31A2 subgroup, telescoping was greater in the OLHS group. This may be partly attributed to slightly better reduction quality in the CMN group, although the difference was not statistically significant. Given that reduction quality strongly influences postoperative stability and telescoping, this factor might have contributed to the observed difference rather than the implant type itself.

This study has limitations. This was a single‐center, retrospective, nonrandomized study. Due to surgeon preference, OLHS was more frequently used for stable AO A1 fractures, whereas CMN was preferred for unstable A2 fractures. This imbalance may affect outcomes such as telescoping and bone union. Specifically, the higher proportion of unstable A2 fractures in the CMN group could have contributed to telescoping values and longer operative times in that cohort. Given the limited sample size, adjusted analyses were not performed; results are presented unadjusted, with descriptive subgroup analyses for A1 and A2 fractures. Larger, randomized, comparative, prospective multicenter studies are needed to consolidate the findings of this study.

## 5. Conclusion

OLHS demonstrated satisfactory outcomes for trochanteric fractures in this exploratory study, with preliminary evidence of adequate stability. Further randomized controlled trials or matched comparative studies are warranted to confirm these findings.

## Ethics Statement

This study was approved by the Institutional Review Board of Shin‐yurigaoka General Hospital (Approval No. 2024‐12‐1, approved on December 2, 2024). All procedures were performed in accordance with the principles of the Declaration of Helsinki.

## Consent

This study was conducted under an opt‐out consent process approved by the ethics committee of Shin‐yurigaoka General Hospital (Approval No. 2024‐12‐1). The study information was disclosed on the institutional website and/or notice boards, and patients were informed that they could withdraw their data from the study at any time. No patients opted out. Written informed consent was obtained from the patient for identification of clinical images (Figures 3(a), 3(b), 3(c), and 3(d)) in this manuscript. Informed consent to participate was provided through an opt‐out method. Information about this study was available to participants (postings in the hospital and on the hospital website) to ensure that they were aware of the study and had an opportunity to opt out if they desired.

## Disclosure

All authors commented on the previous versions of the manuscript and read and approved the final manuscript.

## Conflicts of Interest

Takashi Matsushita is the inventor of a patent related to the Oblique Locking Hip Screw (OLHS) used in this study and receives royalties from HOYA Technosurgical Corporation. Royalties have been paid from 2020 to the present and are ongoing. Takashi Matsushita did not participate in data collection, analysis, or interpretation to minimize potential bias. The other authors declare no conflicts of interest.

## Author Contributions

All authors contributed to the study conception and design. Material preparation, data collection, and analysis were performed by Shota Nakagawa. The first draft of the manuscript was written by Shota Nakagawa.

## Funding

No funding was received for this research.

## Supporting Information

Additional supporting information can be found online in the Supporting Information section.

The following supporting information is available with the online version of this article:

## Supporting information


**Supporting Information 1** Supporting Information 1: Biomechanical testing of the Oblique Locking Hip Screw (OLHS) Static loading, cyclic fatigue, and torsional resistance tests comparing OLHS with conventional CHS and CMN.

**Supporting Information 2** Supporting Information 2: Subgroup analyses includes detailed tables for AO 31A1 (stable), AO 31A2 (unstable), and CMN short vs. long nail comparisons (> Tables S1–S3).

**Supporting Information 3** Supporting Information 3: STROBE checklist for observational cohort studies completed checklist indicating adherence to STROBE reporting guidelines.

## Data Availability

The datasets generated and analyzed during the current study are publicly available in the Zenodo repository: Shota Nakagawa (2025). Dataset of Clinical and Radiographic Outcomes of Trochanteric Femur Fractures Treated with the Oblique Locking Hip Screw [Data set]. Zenodo. https://doi.org/10.5281/zenodo.17375327.
