# Supplementary material for: Clinical Outcome of the Oblique Locking Hip Screw
Source: Adv Orthop. 2025 Nov 17;2025:5082003. doi: 10.1155/aort/5082003 (PMC12621107; doi:10.1155/aort/5082003)
Supplement: Supplementary file 1 — Supporting Information 1 Supporting Information 1: Biomechanical testing of the Oblique Locking Hip Screw (OLHS) Static loading, cyclic fatigue, and torsional resistance tests comparing OLHS with conventional CHS and CMN. [file AORT-2025-5082003-s001.docx]

***Supplementary Material 1. Biomechanical Testing of the Oblique Locking Hip Screw (OLHS)***

All biomechanical tests were performed using an unstable trochanteric fracture model in which the proximal and diaphyseal portions of the femur were not in contact. Comparative evaluations were conducted between the Oblique Locking Hip Screw (OLHS, 3-hole plate) and the conventional Compression Hip Screw (CHS, 4-hole plate), and between the OLHS and the Gamma3 trochanteric nail (with U-lag).

***1. Static Load Test***

Objective: To compare the ultimate axial load-to-failure between OLHS and CHS constructs.

Specimens: Synthetic femoral models (unstable configuration; Sawbones solid rigid polyurethane foam, 40 pcf, ASTM F1839 compliant).

Setup: Each construct was axially loaded to failure at a crosshead speed of 5 mm/min.

| Implant Type | Ultimate Load (N) |
| --- | --- |
| OLHS (3-hole) | 1134 |
| CHS (4-hole) | 960 |

Interpretation: The OLHS demonstrated a higher maximum load-bearing capacity than the conventional CHS, indicating greater construct rigidity under static axial compression.


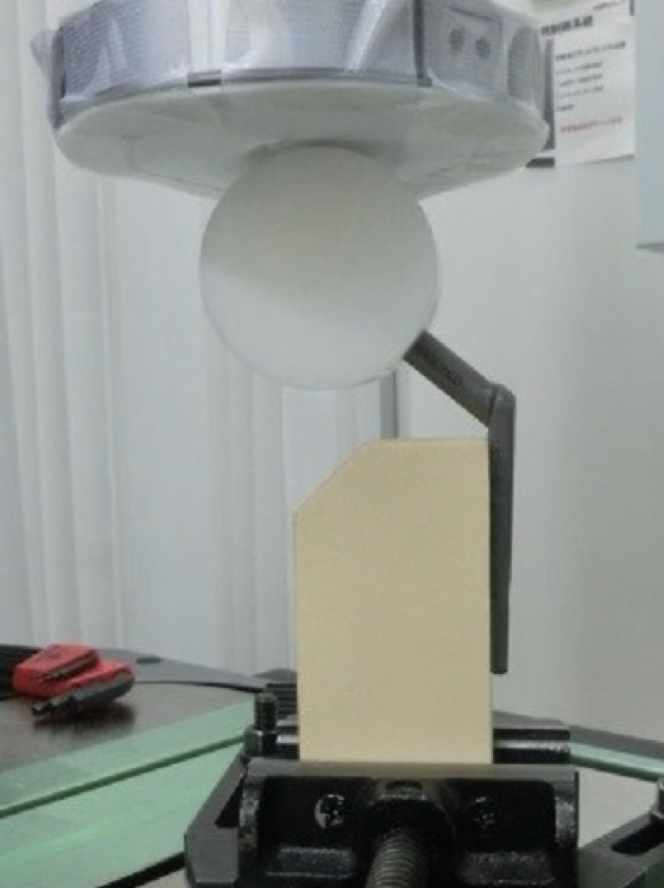


***2. Cyclic Load (Fatigue) Test***

Objective: To compare fatigue resistance between OLHS and CHS under repeated physiological loading.

Testing machine: INSTRON ElectroPuls E1000
Frequency: 3 Hz
Load range: 80 N (min) - 800 N (max)
Specimens: Sawbones solid rigid polyurethane foam (40 pcf, ASTM F1839 compliant). All constructs simulated unstable fracture conditions (no contact between proximal and shaft portions).

| Implant Type | Cycles Endured | Failure Mode |
| --- | --- | --- |
| OLHS (3-hole) | 1,000,000 | No failure |
| CHS (4-hole) | 784 | Distal screw loosening |
| CHS (4-hole) | 584 | Distal screw loosening |

Interpretation: OLHS withstood 1,000,000 cycles at 800 N (3 Hz) without mechanical failure, whereas CHS constructs failed after approximately 584 - 784 cycles due to distal screw loosening. These results confirm the superior fatigue strength of the OLHS system under cyclic axial loading.

***3. Anti-rotational Stability Test (Static Loading)***

Objective: To assess the torsional stability of the proximal fragment fixation compared with a cephalomedullary nail (Gamma3, U-lag).

Setup: The proximal fragment was loaded 100 mm from the femoral head center at a crosshead speed of 5 mm/min. Load–displacement curves were recorded until the loading part detached or displacement reached 16.7 mm.

Results: OLHS exhibited markedly higher rotational stiffness and load resistance than Gamma3. At the 16.7 mm displacement endpoint, OLHS maintained substantially higher torque and rigidity (see Fig. S2).

Interpretation: The oblique multi-pin locking design of OLHS provides enhanced rotational resistance compared with Gamma3 under static torsional conditions.


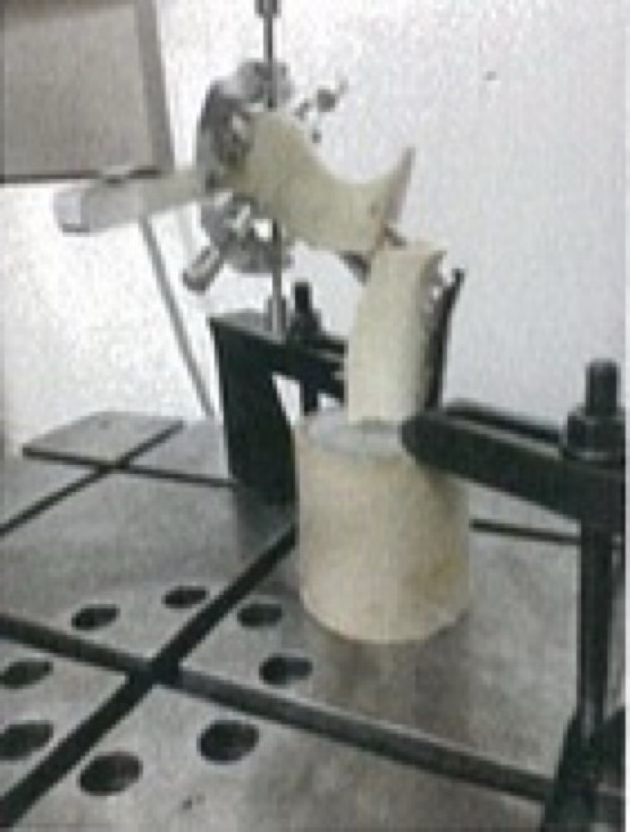


Figure S2a:

Figure S2b:

***4. Anti-rotational Stability Test (Cyclic Loading)***

Objective: To evaluate torsional endurance under repeated cyclic loading.
Testing conditions: Torque range: +1 Nm to −1 Nm; Frequency: 1 Hz; Endpoint: 20° rotation of the proximal fragment.

| Implant Type | Cycles to 20° Rotation |
| --- | --- |
| OLHS | >900,000 (no failure) |
| Gamma3 (U-lag) | 151 |
| Gamma3 (U-lag) | 217 |
| Gamma3 (U-lag) | 135 |

Interpretation: The OLHS maintained rotational stability for more than 900,000 cycles without measurable failure, whereas the Gamma3 constructs reached 20° rotation between 135 and 217 cycles. OLHS therefore demonstrated substantially higher torsional endurance than the intramedullary nail construct under cyclic conditions.


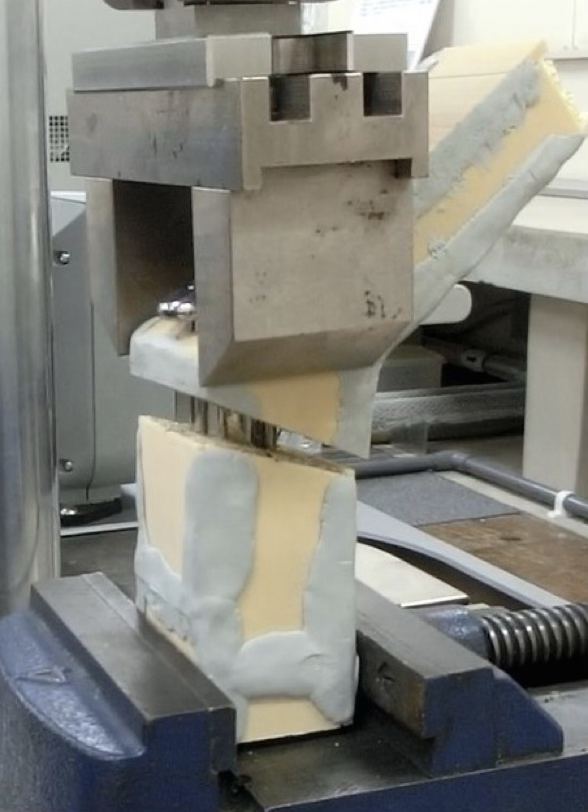


Figure

***Summary of Biomechanical Tests***

| Test Type | Comparison | Key Finding | Interpretation |
| --- | --- | --- | --- |
| Static axial load | OLHS vs CHS | 1134 vs 960 N | OLHS higher static rigidity |
| Cyclic axial load | OLHS vs CHS | 1,000,000 vs 784 / 584 cycles | OLHS superior fatigue resistance |
| Static torsion | OLHS vs Gamma3 | OLHS > Gamma3 | OLHS higher rotational stiffness |
| Cyclic torsion | OLHS vs Gamma3 | >900,000 vs 151 / 217 / 135 cycles | OLHS markedly superior torsional endurance |

Conclusion: Under identical unstable fracture conditions, the Oblique Locking Hip Screw (OLHS) demonstrated superior static strength, fatigue endurance, and rotational stability compared with both conventional CHS and Gamma3 intramedullary nail constructs. These biomechanical advantages support its clinical use for stable and selected unstable trochanteric fractures.
