# Supplementary material for: Clinical Outcome of the Oblique Locking Hip Screw
Source: Adv Orthop. 2025 Nov 17;2025:5082003. doi: 10.1155/aort/5082003 (PMC12621107; doi:10.1155/aort/5082003)
Supplement: Supplementary file 2 — Supporting Information 2 Supporting Information 2: Subgroup analyses includes detailed tables for AO 31A1 (stable), AO 31A2 (unstable), and CMN short vs. long nail comparisons (> Tables S1–S3). [file AORT-2025-5082003-s002.docx]

***Supplementary Material 2. Subgroup Analysis***

**Table S1. Outcomes in AO 31A1 fractures (stable type)**

Comparison of demographic, radiographic, and clinical outcomes between OLHS and CMN in stable (A1) fractures.

|  | | OLHS | CMN |  | difference |
| --- | --- | --- | --- | --- | --- |
|  | | n=33 | n=14 | p | [95% CI] |
| ***Background data*** | |  |  |  |  |
| Age (mean) | | 86.6±8.7 | 85.1±8.4 | 0.60* | 1.5 [−4.1, 7.1] |
| Sex (Male/Female) | | 9/24 | 4/10 | 1.0** |  |
| BMI | | 19.0±2.9 | 20.9±2.9 | 0.06* | -1.9 [−3.8, 0.1] |
| Follow-up period (month) | |  |  |  |  |
|  | Clinical | 32.5±18.1 | 33.1±22.6 | 0.93* | -0.6 [−14.8, 13.6] |
|  | Radiographic | 18.1±15.5 | 20.2±21.6 | 0.75* | -2.1 [−15.4, 11.3] |
| Operating time (min) | | 65.6±25.9 | 71.6±41.7 | 0.62* | -6.0 [−31.3, 19.3] |
| Blood loss (cc) | | 38.9±47.7 | 71.9±183.8 | 0.52* | -33.0 [−140.0, 74.0] |
| ASA PS | |  |  | 1.0** |  |
|  | PS 1 | 0 | 0 |  |  |
|  | PS 2 | 20 | 9 |  |  |
|  | PS 3 | 13 | 5 |  |  |
| ***Fracture type*** | |  |  |  |  |
| Tang classification | |  |  | 0.30** |  |
|  | Type 1 | 33 | 13 |  |  |
|  | Type 2 | 0 | 1 |  |  |
|  | Type 3 | 0 | 0 |  |  |
|  | Type 4 | 0 | 0 |  |  |
|  | Type 5 | 0 | 0 |  |  |
| Nakano 3DCT classification | |  |  | 0.24** |  |
|  | Type 1-2 | 25 | 13 |  |  |
|  | Type 1-3 | 8 | 1 |  |  |
|  | Type 1-4 | 0 | 0 |  |  |
|  | Type 2 | 0 | 0 |  |  |
| *Mann-Whitney U test  **Fisher’s Exact Test  OLHS, oblique locking hip screw; CMN, cephalomedullary nail; CI, confidence interval; BMI, body mass index; ASA PS, American Society of Anesthesia Physical Status | | | | | |

|  | | | OLHS  n=33 | | CMN  n=14 | | p | | difference  [95% CI] |
| --- | --- | --- | --- | --- | --- | --- | --- | --- | --- |
| ***Postoperative assessment*** | | |  | |  | |  | |  |
| Utsunomiya classification | | |  | |  | | 1.0** | |  |
|  | Intramedullary | | 2 | | 1 | |  | |  |
|  | Extramedullary valgus | | 31 | | 13 | |  | |  |
|  | Extramedullary varus | | 0 | | 0 | |  | |  |
| Ikuta classification | | |  | |  | | 0.79** | |  |
|  | Subtype A | | 7 | | 4 | |  | |  |
|  | Subtype N | | 22 | | 9 | |  | |  |
|  | Subtype P | | 4 | | 1 | |  | |  |
| Baumgaertner classification | | |  | |  | | 0.63** | |  |
|  | Good | | 30 | | 12 | |  | |  |
|  | Acceptable | | 3 | | 2 | |  | |  |
|  | Poor | | 0 | | 0 | |  | |  |
| Chang classification | | |  | |  | | 1.0** | |  |
|  | Excellent | | 28 | | 12 | |  | |  |
|  | Acceptable | | 5 | | 2 | |  | |  |
|  | Poor | | 0 | | 0 | |  | |  |
| Tip–apex distance | | |  | |  | | 0.30** | |  |
|  | >25 mm | | 0 | | 1 | |  | |  |
|  | ≦25 mm | | 33 | | 13 | |  | |  |
| Telescoping (mm) | | | 4.1±4.0 | | 3.1±6.5 | | 0.63* | | 0.92 [-3.0, 4.9] |
| *Mann-Whitney U test  **Fisher’s Exact Test  OLHS, oblique locking hip screw; CMN, cephalomedullary nail; CI, confidence interval; | | | | | | | | | |
|  | | OLHS  n=33 | | CMN  n=14 | | p | |  |  |
| ***Clinical outcomes*** | |  | |  | |  | |  |  |
| Mortality | ≦1 month | 1 | | 0 | | 1.0* | |  |  |
|  | ≦3 month | 2 | | 0 | | 1.0* | |  |  |
| Clinical outcomes at the last visit | |  | |  | |  | |  |  |
| Bone union | | 31 | | 13 | | 1.0* | |  |  |
| Non-union | | 0 | | 1 | | 0.30* | |  |  |
| Cut-through | | 0 | | 0 | | - | |  |  |
| *Fisher’s Exact Test | |  | |  | |  | |  |  |

OLHS, oblique locking hip screw; CMN, cephalomedullary nail; CI, confidence interval;

**Table S2. Outcomes in AO 31A2 fractures (unstable type)**

Subgroup analysis of unstable (A2) fractures comparing OLHS and CMN.

|  | | OLHS | CMN |  | difference |
| --- | --- | --- | --- | --- | --- |
|  | | n=24 | n=43 | p | [95% CI] |
| ***Background data*** | |  |  |  |  |
| Age (mean) | | 85.4±8.1 | 84.6±9.8 | 0.74* | 2.2 [−3.7, 5.2] |
| Sex (Male/Female) | | 4/20 | 5/38 | 0.71** |  |
| BMI | | 20.6±3.6 | 20.5±3.8 | 0.91* | -0.1 [−1.8, 2.0] |
| Follow-up period (month) | |  |  |  |  |
|  | Clinical | 30.0±16.8 | 40.1±21.0 | 0.04* | -10.0 [−19.4, -0.1] |
|  | Radiographic | 13.2±11.5 | 17.9±14.0 | 0.15* | -4.6 [−11.0, 1.7] |
| Operating time (min) | | 84.5±21.3 | 81.7±50.3 | 0.75* | -2.8 [−18.8, 24.3] |
| Blood loss (cc) | | 105.7±117.6 | 87.2±125.9 | 0.55* | 18.5 [−43.2, 80.2] |
| ASA PS | |  |  | 0.41** |  |
|  | PS 1 | 0 | 3 |  |  |
|  | PS 2 | 18 | 31 |  |  |
|  | PS 3 | 6 | 9 |  |  |
| ***Fracture type*** | |  |  |  |  |
| Tang classification | |  |  | 0.90** |  |
|  | Type 1 | 7 | 12 |  |  |
|  | Type 2 | 0 | 1 |  |  |
|  | Type 3 | 10 | 18 |  |  |
|  | Type 4 | 7 | 12 |  |  |
|  | Type 5 | 0 | 0 |  |  |
| Nakano 3DCT classification | |  |  | 0.57** |  |
|  | Type 1-2 | 0 | 0 |  |  |
|  | Type 1-3 | 19 | 31 |  |  |
|  | Type 1-4 | 5 | 12 |  |  |
|  | Type 2 | 0 | 0 |  |  |
| *Mann-Whitney U test  **Fisher’s Exact Test  OLHS, oblique locking hip screw; CMN, cephalomedullary nail; CI, confidence interval; BMI, body mass index; ASA PS, American Society of Anesthesia Physical Status | | | | | |

|  | | | OLHS  n=24 | | CMN  n=43 | | p | | difference  [95% CI] |
| --- | --- | --- | --- | --- | --- | --- | --- | --- | --- |
| ***Postoperative assessment*** | | |  | |  | |  | |  |
| Utsunomiya classification | | |  | |  | | 0.56** | |  |
|  | Intramedullary | | 0 | | 1 | |  | |  |
|  | Extramedullary valgus | | 24 | | 41 | |  | |  |
|  | Extramedullary varus | | 0 | | 1 | |  | |  |
| Ikuta classification | | |  | |  | | 0.02** | |  |
|  | Subtype A | | 12 | | 10 | |  | |  |
|  | Subtype N | | 8 | | 30 | |  | |  |
|  | Subtype P | | 4 | | 3 | |  | |  |
| Baumgaertner classification | | |  | |  | | 0.14** | |  |
|  | Good | | 19 | | 39 | |  | |  |
|  | Acceptable | | 3 | | 4 | |  | |  |
|  | Poor | | 2 | | 0 | |  | |  |
| Chang classification | | |  | |  | | 0.36** | |  |
|  | Excellent | | 19 | | 39 | |  | |  |
|  | Acceptable | | 4 | | 4 | |  | |  |
|  | Poor | | 1 | | 0 | |  | |  |
| Tip–apex distance | | |  | |  | | - | |  |
|  | >25 mm | | 0 | | 0 | |  | |  |
|  | ≦25 mm | | 24 | | 43 | |  | |  |
| Telescoping (mm) | | | 5.8±4.6 | | 2.9±4.1 | | 0.013* | | 2.9 [0.7, 5.2] |
| *Mann-Whitney U test  **Fisher’s Exact Test  OLHS, oblique locking hip screw; CMN, cephalomedullary nail; CI, confidence interval; | | | | | | | | | |
|  | | OLHS  n=24 | | CMN  n=43 | | p | |  |  |
| ***Clinical outcomes*** | |  | |  | |  | |  |  |
| Mortality | ≦1 month | 0 | | 0 | | - | |  |  |
|  | ≦3 month | 0 | | 1 | | 1.0* | |  |  |
| Clinical outcomes at the last visit | |  | |  | |  | |  |  |
| Bone union | | 23** | | 39 | | 1.0* | |  |  |
| Non-union | | 0 | | 2 | | 1.0* | |  |  |
| Cut-through | | 0 | | 1 | | 1.0* | |  |  |
| *Fisher’s Exact Test  ** **One patient who was followed for more than 3 months died before radiographic bone union was confirmed.**  **This case was excluded from the bone union analysis but was not classified as non-union.** | | | | | | | |  |  |

OLHS, oblique locking hip screw; CMN, cephalomedullary nail

**Table S3. Comparison between short and long CMN constructs**

Exploratory comparison of clinical and radiographic parameters between short and long cephalomedullary nail constructs.

|  | | | SFN | | | LFN | | |  | | | difference | |  |
| --- | --- | --- | --- | --- | --- | --- | --- | --- | --- | --- | --- | --- | --- | --- |
|  | | | n=30 | | | n=27 | | | p | | | [95% CI] | |  |
| ***Background data*** | | |  | | |  | | |  | | |  | |  |
| Age (mean) | | | 86.5±9.3 | | | 82.8±9.4 | | | 0.14* | | | 3.8 [−1.2, 8.7] | |  |
| Sex (Male/Female) | | | 4/26 | | | 5/22 | | | 0.72** | | |  | |  |
| BMI | | | 20.6±3.8 | | | 20.6±3.3 | | | 0.99* | | | -0.02 [−1.9, 1.9] | |  |
| Follow-up period (month) | | |  | | |  | | |  | | |  | |  |
|  | | Clinical | 39.3±21.8 | | | 37.3±21.3 | | | 0.72* | | | 2.0 [−9.4, 13.5] | |  |
|  | | Radiographic | 19.6±16.1 | | | 17.1±16.2 | | | 0.55* | | | 2.6 [−6.0, 11.2] | |  |
| Operating time (min) | | | 67.6±38.2 | | | 92.2±55.1 | | | 0.06* | | | -24.5 [−50.1, 1.0] | |  |
| Blood loss (cc) | | | 52.1±127.9 | | | 118.3±148.2 | | | 0.08* | | | -66.2 [−140.2, 7.8] | |  |
| ASA PS | | |  | | |  | | | 0.75** | | |  | |  |
|  | | PS 1 | 1 | | | 2 | | |  | | |  | |  |
|  | | PS 2 | 21 | | | 19 | | |  | | |  | |  |
|  | | PS 3 | 8 | | | 6 | | |  | | |  | |  |
| ***Fracture type*** | | |  | | |  | | |  | | |  | |  |
| AO classification | | |  | | |  | | | 0.005** | | |  | |  |
|  | | 31A1 | 12 | | | 2 | | |  | | |  | |  |
|  | | 31A2 | 18 | | | 25 | | |  | | |  | |  |
| Tang classification | | |  | | |  | | | <0.001** | | |  | |  |
|  | | Type 1 | 21 | | | 4 | | |  | | |  | |  |
|  | | Type 2 | 1 | | | 1 | | |  | | |  | |  |
|  | | Type 3 | 7 | | | 11 | | |  | | |  | |  |
|  | | Type 4 | 1 | | | 11 | | |  | | |  | |  |
|  | | Type 5 | 0 | | | 0 | | |  | | |  | |  |
| Nakano 3DCT classification | | |  | | |  | | | 0.003** | | |  | |  |
|  | | Type 1-2 | 11 | | | 2 | | |  | | |  | |  |
|  | | Type 1-3 | 17 | | | 15 | | |  | | |  | |  |
|  | | Type 1-4 | 2 | | | 10 | | |  | | |  | |  |
|  | | Type 2 | 0 | | | 0 | | |  | | |  | |  |
| ***Implant information*** | | |  | | |  | | | 0.13** | | |  | |  |
| TFN Advanced | |  |  | | |  | | |  | | |  | |  |
|  | | Blade | 6 | | | 4 | | |  | | |  | |  |
|  | | Screw | 4 | | | 0 | | |  | | |  | |  |
|  | | Cement | 1 | | | 0 | | |  | | |  | |  |
| InterTAN | |  | 19 | | | 23 | | |  | | |  | |  |
| *Mann-Whitney U test  **Fisher’s Exact Test  SFN, short femoral nail; LFN, long femoral nail; CI, confidence interval; BMI, body mass index; ASA PS, American Society of Anesthesia Physical Status | | | | | | | | | | | | | |  |
|  | | | | | SFN  n=30 | | LFN  n=27 | | | p | | | difference  [95% CI] | |
| ***Postoperative assessment*** | | | | |  | |  | | |  | | |  | |
| Utsunomiya classification | | | | |  | |  | | | 0.57** | | |  | |
|  | Intramedullary | | | | 1 | | 1 | | |  | | |  | |
|  | Extramedullary valgus | | | | 29 | | 25 | | |  | | |  | |
|  | Extramedullary varus | | | | 0 | | 1 | | |  | | |  | |
| Ikuta classification | | | | |  | |  | | | 0.93** | | |  | |
|  | Subtype A | | | | 8 | | 6 | | |  | | |  | |
|  | Subtype N | | | | 20 | | 19 | | |  | | |  | |
|  | Subtype P | | | | 2 | | 2 | | |  | | |  | |
| Baumgaertner classification | | | | |  | |  | | | 0.09** | | |  | |
|  | Good | | | | 29 | | 22 | | |  | | |  | |
|  | Acceptable | | | | 1 | | 5 | | |  | | |  | |
|  | Poor | | | | 0 | | 0 | | |  | | |  | |
| Chang classification | | | | |  | |  | | | 1.0** | | |  | |
|  | Excellent | | | | 27 | | 24 | | |  | | |  | |
|  | Acceptable | | | | 3 | | 3 | | |  | | |  | |
|  | Poor | | | | 0 | | 0 | | |  | | |  | |
| Tip–apex distance | | | | |  | |  | | | 1.0** | | |  | |
|  | >25 mm | | | | 1 | | 0 | | |  | | |  | |
|  | ≦25 mm | | | | 29 | | 27 | | |  | | |  | |
| Telescoping (mm) | | | | | 2.5±4.7 | | 3.5±4.8 | | | 0.40* | | | -1.1 [-3.6, 1.4] | |
| *Mann-Whitney U test  **Fisher’s Exact Test  SFN, short femoral nail; LFN, long femoral nail; CI, confidence interval | | | | | | | | | | | | | | |
|  | | | | SFN  n=30 | | LFN  n=27 | | p | | |  |  |  |  |
| ***Clinical outcomes*** | | | |  | |  | |  | | |  |  |  |  |
| Mortality | ≦1 month | | | 0 | | 0 | | - | | |  |  |  |  |
|  | ≦3 month | | | 0 | | 1 | | 0.47* | | |  |  |  |  |
| Clinical outcomes at the last visit | | | |  | |  | |  | | |  |  |  |  |
| Bone union | | | | 29 | | 24 | | 0.34* | | |  |  |  |  |
| Non-union | | | | 1 | | 1 | | 1.0* | | |  |  |  |  |
| Cut-through | | | | 0 | | 1 | | 0.47* | | |  |  |  |  |
| *Fisher’s Exact Test | | | |  | |  | |  | | |  |  |  |  |

SFN, short femoral nail; LFN, long femoral nail
