# Supplementary material for: Clinical Outcome of the Oblique Locking Hip Screw
Source: Adv Orthop. 2025 Nov 17;2025:5082003. doi: 10.1155/aort/5082003 (PMC12621107; doi:10.1155/aort/5082003)
Supplement: Supplementary file 3 — Supporting Information 3 Supporting Information 3: STROBE checklist for observational cohort studies completed checklist indicating adherence to STROBE reporting guidelines. [file AORT-2025-5082003-s003.docx]

**Supplementary Material 3**

**STROBE Statement—Checklist for Observational Studies (Cohort Study)**

This checklist refers to the study titled “Clinical outcome of the Oblique Locking Hip Screw”. It is a retrospective single-center observational cohort study conducted at Shin-yurigaoka General Hospital between April 2020 and December 2022. The study evaluated clinical outcomes of the Oblique Locking Hip Screw (OLHS) compared with Cephalomedullary Nail (CMN) fixation for trochanteric femoral fractures. The main outcome was bone union rate, with secondary outcomes including operative time, blood loss, cut-through, mortality, and telescoping.

| Item No. | STROBE Item | Recommendation | Location in Manuscript |
| --- | --- | --- | --- |
| 1 | Title and abstract | Indicate study design with a common term in the title or abstract. | Title and Abstract – clearly state 'retrospective single-center study'. |
| 2 | Background/rationale | Explain the scientific background and rationale. | Introduction, paragraphs 1–3. |
| 3 | Objectives | State specific objectives and hypotheses. | Introduction, final paragraph. |
| 4 | Study design | Present key design elements early. | Methods – Study design paragraph. |
| 5 | Setting | Describe setting, location, and relevant dates. | Methods – Study design and patient selection. |
| 6 | Participants | Eligibility criteria and methods of selection. | Methods – Inclusion/exclusion criteria. |
| 7 | Variables | Define outcomes, exposures, predictors, confounders. | Methods – Postoperative Assessment. |
| 8 | Data sources/measurement | Provide data sources and measurement details. | Methods – Radiographic Measurements. |
| 9 | Bias | Describe efforts to address bias. | Discussion – Limitations (selection bias). |
| 10 | Study size | Explain how sample size was determined. | Methods – Statistical Analyses. |
| 11 | Quantitative variables | Explain handling of quantitative variables. | Methods – Statistical Analyses. |
| 12 | Statistical methods | Describe all statistical methods and confounder control. | Methods – Statistical Analyses (Fisher, Mann–Whitney, 95% CI, two-tailed). |
| 13 | Participants (Results) | Report participant numbers and exclusions. | Results – Flowchart (Figure 1). |
| 14 | Descriptive data | Provide characteristics of participants. | Results – Patient Demographics (Table 1). |
| 15 | Outcome data | Report outcomes or summary measures. | Results – Clinical Outcomes (Table 3, S1–S3). |
| 16 | Main results | Provide unadjusted/adjusted estimates with precision. | Results – All tables include p-values and 95% CI. |
| 17 | Other analyses | Report subgroup analyses and interactions. | Results – Subgroup Analyses (A1, A2, CMN short/long). |
| 18 | Key results | Summarize key results referencing objectives. | Discussion – Opening paragraphs. |
| 19 | Limitations | Discuss limitations and bias. | Discussion – Limitations section. |
| 20 | Interpretation | Give cautious interpretation of results. | Discussion – Exploratory tone, no overstatement. |
| 21 | Generalisability | Discuss external validity of results. | Discussion – Applicability to similar populations, multicenter need. |
| 22 | Funding | Give funding sources and role of funders. | Declarations – Funding: none; COI disclosed; independent analysis. |
